# Everyday drug diversions: A qualitative study of the illicit exchange and non-medical use of prescription stimulants on a university campus

**DOI:** 10.1016/j.socscimed.2014.10.016

**Published:** 2015-04

**Authors:** Scott Vrecko

**Affiliations:** Department of Social Science, Health and Medicine, King's College London, Strand, London WC2R 2LS, UK

**Keywords:** United States, Pharmaceuticals, Drug diversion, Pharmaceuticalization, Non-medical drug use, Prescription medication, Enhancement, Adderall

## Abstract

This article investigates everyday experiences and practises that are associated with processes of pharmaceuticalization and with practices of ‘drug diversion’—that is, the illicit exchange and non-medical use of prescription drugs. It reports results from a qualitative study that was designed to examine the everyday dimensions of non-medical prescription stimulant use among students on an American university campus, which involved 38 semi-structured interviews with individuals who used prescription stimulants as a means of improving academic performance. While discussions of drug diversion are often framed in terms of broad, population-level patterns and demographic trends, the present analysis provides a complementary sociocultural perspective that is attuned to the local and everyday phenomena. Results are reported in relation to the acquisition of supplies of medications intended for nonmedical use. An analysis is provided which identifies four different sources of diverted medications (friends; family members; black-market vendors; deceived clinicians), and describes particular sets of understandings, practices and experiences that arise in relation to each different source. Findings suggest that at the level of everyday experience and practice, the phenomenon of prescription stimulant diversion is characterised by a significant degree of complexity and heterogeneity.

## Highlights

• Reports original research on non-medical stimulant use among university students.
• Explores mechanisms of drug diversion from the perspective of everyday users.
• Offers empirical insight on local organisation of illicit pharmaceutical markets.
• Furthers understandings of everyday dimensions of ‘pharmaceuticalization’.

## 1 Introduction

ALEXIS: We were in the same class; she lived down the hall, so we worked a lot together. I was done, but she wanted me to stay. She was actually wanting me to study with her, and I was just, ‘I'm tired’. It doesn't take me that long to do my part, and she had a little bit more trouble, and it took her a little bit longer. So she was, ‘all right, well—you can take an Adderall and just stay up with me in the library!’ And at first, when she said it, I was: ‘no, I'm not taking Adderall. I'm just going to go to sleep, and I'll help you tomorrow.’

Because of the phobia of its being a pill, you automatically assume that you're going to get an out-of-body experience, you're doing something wrong, and it's just … I've never been prescribed anything, so it just didn't feel right. But then on the other hand, I didn't have any hesitation [in relation to concerns about whether taking the pill] would hurt me because I knew so many other people that were taking it at time, more frequently. So I just was, ‘all right, I’ll help you out’, and popped it. I ended up taking it just because she was really struggling in the class. And once I took it, I was, ‘oh, this isn't that bad.’ And it didn't bother me, and I didn't feel like I was doing anything wrong.

Alexis is a third-year undergraduate science major who regularly uses Adderall—a stimulant medication composed of mixed amphetamine salts that is produced by Shire Pharmaceuticals and approved for use in the treatment of Attention Deficit and Hyperactivity Disorder (ADHD) and narcolepsy—to help her study more effectively. Above, she is telling me about how she came to try the drug for the first time, during a study session with one of her classmates. After several hours of studying, Alexis had finished her work, was tired, and was ready to call it a day; however, when her friend offers her a tablet of Adderall to help her get over any tiredness she might feel, Alexis accepts the offer. And though she tells me that at the time she considered herself to be someone who ‘would never do that sort of thing’, she subsequently begins to use the pills as regularly as she can obtain them, which she does by ‘dropping hints’ to her friend.

Alexis's non-medical use of Adderall is part of a trend that has been identified by a range of scholars: the increasing use of pharmaceuticals approved for therapeutic purposes as a means to enhance the mental capacities of ‘normal’ individuals, i.e. those who are not ill (Kramer, 1992; Parens, 1998; Quintero and Nichter, 2011). While a wide range of prescription medications are consumed for unapproved, non-therapeutic purposes, the use of stimulant medications by individuals—particularly researchers and university students—seeking to boost their abilities to concentrate and focus on academic work has become one of the main areas of focus within discussions of enhancement (Arria, 2008; Elnicki, 2013; Maher, 2008). This phenomenon raises a number of ethical and policymaking questions that have received attention from bioethicists, such as whether pharmaceutical enhancement constitutes a form of cheating and whether individuals who do not use pharmaceutical enhancers might experience coercion (S. Bell et al., 2013; Farah et al., 2004; Greely et al., 2008; Rudski, 2014). It also raises significant issues that have been examined by social scientists, from political–economic concerns about the scientific and commercial choices involved in the development of medications that blur the boundary between normal and pathological cognition (Bond, 2009; Moreira et al., 2009; Whitehouse et al., 2005), to transformations in medical practice and patient expectations associated with increasing medicalization (Conrad and Leiter, 2004; Coveney et al., 2011).

A growing body of empirical research provides useful insight into non-medical prescription stimulant use, particularly in the form of regional and national surveys that examine broad demographic patterns and social attitudes (Hotze et al., 2011; Maher, 2008; Pilkinton and Cannatella, 2012; Teter et al., 2006). In the US context, significant levels of non-medical prescription stimulant use have been found among university students, with estimates of lifetime prevalence rates as high as 6.9% (McCabe et al., 2005). At the same time, variations have been reported across different regions of the country and among different groups of academic institutions: for example, lifetime prevalence rates of non-medical prescription stimulant use are reported to be as high as 25% at institutions located in the American north-east, with more competitive admissions standards (Loe, 2008; Wilens et al., 2008). Further variations appear among student subpopulations, with greater prevalence of use among white male students, and members of fraternities and sororities (Hall et al., 2005; McCabe et al., 2005).

Survey findings such as those above are crucial for revealing significant demographic and historical trends about non-medical prescription stimulant use. However, they are limited in their ability to produce detailed information on the lived experiences and daily practices of actual users (Loe and Cuttino, 2008; Singh et al., 2010). It is increasingly recognized that qualitative research has the capacity to offer distinctive insight in this respect (Lucke, 2012), and although empirical data on the experiences and practices of everyday users remains somewhat limited (Singh and Kelleher, 2010; Varga, 2012), findings from several studies have been published in recent years (Loe, 2008; Loe and Cuttino, 2008; Partridge et al., 2013; Vrecko, 2013). This article aims to contribute to this emerging body of work, presenting findings from a qualitative study designed to further understandings of the everyday dimensions of non-medical prescription stimulant use among university students seeking to improve their academic performance.

### 1.1 Pharmaceutical leakage: drug diversion and everyday life

Qualitative research on prescription stimulant use among university students to date has largely focused on exploring the subjective views and experiences of this population. For example, Loe and Cuttino (2008)'s study of students diagnosed with ADHD focuses on forms of identity management and self-conception associated with medication use, while Partridge et al. (2013) examine perceptions of drug efficacy and safety, and Vrecko (2013) reports on non-medical users' accounts of experiences of studying while on prescription stimulants. This article takes a rather different focus, however, exploring processes associated with prescription drug ‘diversion’, that is, the movement of medications away from those to whom they have been prescribed legally, to those who obtain and use them illegally and for non-therapeutic purposes.

In recent decades, the non-medical use of prescription drugs has been identified as a significant and growing phenomenon, and has received increasing attention from public health experts, medical practitioners, and government agencies involved in law enforcement and drug abuse prevention. While there is some emerging data on national trends in stimulant drug diversion (McCabe et al., 2014; Varga, 2012), relatively little is known about the transactions that arise as prescription stimulants enter into and circulate within networks of non-medical users (Fischer et al., 2010; Wilens et al., 2008). Much of existing research and commentary relating to drug diversion has been oriented towards population-level analyses that are linked to forms of epidemiologic inquiry, and survey-based data findings. In comparison, relatively few studies have explored non-medical prescription drug use and processes of drug diversion in terms of the smaller-scale social and interpersonal dynamics underlying these broad patterns of consumption. The present analysis is based on the hypothesis that fine-grained sociocultural approaches may be valuable for understanding the local particularities and processes from which population-level trends arise (Quintero et al., 2006).

An example of such a socio-cultural approach is provided by the anthropologist Anne Lovell and her ethnographic analysis of the ‘pharmaceutical leakage’ (2006) that arises in relation to buprenorphine diversion in France. While predominantly oriented toward the specific case of buprenorphine use and exchange among her informants, Lovell's study may be taken—as it is here—as a starting point for considering how patterns of national drug diversion can be investigated at the micro level as a social and interactive phenomenon. For example, Lovell suggests that pharmaceutical diversion might be described as a process that ‘connects the doctor's office or the pharmacy with networks of drug users who can diffuse the product and knowledge about it’ (2006: 156). While Lovell fully recognizes that local actors' activities are tied to broad cultural dynamics and political-economic systems, one of her most crucial findings is that macro-level patterns cannot be fully understood without an account of the everyday beliefs and actions of individuals who seek, receive, and distribute diverted pharmaceuticals: the different practices and strategies associated with acquiring medications, as well as the intersubjective understandings and local knowledges that circulate with them. The novelty of such a perspective, in comparison to many other analyses of drug diversion, is that it places emphasis on developing an empirical account of the everyday practices that arise as pharmaceutical products ‘leak’ out from the legalised flows of industrial production and medically-condoned distribution, to the illicit realm of black-market distribution and non-medical use.

The analysis below takes inspiration from sociocultural analyses such as Lovell's insofar as it approaches drug diversion as a social process to be explained, rather than as an epidemiological pattern to be measured. Exploring the mechanisms through which prescription psychostimulants are acquired and exchanged among students on an elite American university campus, it focuses in particular on what Lovell describes as ‘the pharmaco-associative’, that is, ‘the indigenous transmission and elaboration of knowledge about psychoactive substances and the ongoing interaction and ensuing social organization of the drug users themselves’ (2006: 156). After providing an account of research methods and data analysis, it presents results which may be considered as representations of different forms of ‘pharmaco-association’ that appear among users, as these relate to individuals' strategies for obtaining Adderall for illicit, non-medical purposes. A subsequent discussion further considers how the everyday circumstances, shared meanings and social practices that are reported may be linked to ‘translocal’ processes, including those associated with contemporary configurations of medical knowledge and clinical practice, national regulatory apparatuses, and commercial pharmaceutical enterprise.

Beyond developing insights on the specific case of illicit, nonmedical use of prescription stimulants, a subsidiary aim of the current paper is to contribute to fine-grained, micro-level studies of ‘pharmaceuticalization’ that explore the social, personal and everyday dynamics that arise as pharmaceutical products come to be put to work in the management of an increasingly wide array of problems and experiences. In line with contemporary empirically-oriented approaches, pharmceuticalization is understood here as a ‘dynamic and complex heterogeneous socio-technical process’ (Williams et al., 2011: 721) linked to increasing rates of pharmaceutical consumption (Bell and Figert, 2012; Busfield, 2010); and I suggest that qualitative approaches attuned to everyday consumer practices hold significant potential for contributing to understandings of how individuals and consumer experiences intersect with broader political and economic dynamics (Fox et al., 2005; Jones, 2008; Stevenson et al., 2008).

### 1.2 Methods and data analysis

Findings presented here were generated from a qualitative investigation conducted by the author over a three-year period (2009–2011). Data was collected over 5 months of fieldwork at an elite US university, involving thirty-eight semi-structured interviews designed to elicit informal conversation about the beliefs, practices, and experiences of non-medical users (17 women, 21 men) of prescription stimulants. Initial subjects were recruited through posters placed in student areas on the university campus, and through a call for participants circulated via an email listserv run by the university; snowball sampling was also subsequently used, as participants informed friends, acquaintances, and colleagues about the study. Research and consent procedures were reviewed and approved by the university's IRB.

Recruitment materials requested participants who had experience using Ritalin, Adderall, or other medications as ‘study aids’, and to be included in the dataset used for the following analysis participants had to: (1) be a former or current university student; (2) have experience using prescription stimulants as a means of improving academic performance, over a period of at least 3 months; and (3) not self-identify as having ADHD or any other psychiatric condition associated with impaired academic performance. Interviews were recorded and transcribed in full, and transcripts were imported into the qualitative analysis software Nvivo (Bazeley, 2007). Consonant with a grounded theory approach (Charmaz, 2006), data were coded and analytic induction used to identify key themes. Informants reported using a number of different medications, including Ritalin, Concerta, and Dexedrine, but the analysis below focuses on one particular product—namely Adderall—as this was what the overwhelming majority of subjects reported experiences with. In order to ensure anonymity, pseudonyms have been used for all informants' names.

## 2 Results

As a result of preliminary analysis of data, variation in the sources from which individuals obtained Adderall was identified as a significant theme, relating to differences in informants' practises and experiences with the drug. Subsequent analysis identified four different sources, each of which was in turn associated with different ‘acquisition strategies’ that individuals employed in order to obtain medication for non-therapeutic use. Two of the strategies involved gift-like exchanges of Adderall, in which medication was obtained from friends or family members without any financial payment. Another two strategies involved the procurement of Adderall in relation to commercial transactions, either through involvement in local black markets, or by accessing legal pharmaceutical markets after having fraudulently obtained a doctor's prescription. Every one of the individuals interviewed reported experiences relating to one or more of these four themes.

### 2.1 Acquisition from friends

The most common way that informants reported obtaining Adderall for non-medical use involved receiving pills from someone known personally to them, and well enough to be described as a friend. More than three-quarters of individuals (n29) reported such transactions, in which a recipient would typically be given a small supply of pills without expectation of a monetary payment or other financial exchange. Such gifts would usually, although not always, come from individuals who themselves had a prescription for the medication; and they would normally consist of no more than a few pills—often, only one or two which would be expected to be consumed on a single occasion.

The fact that pills were provided only a few at a time meant that regular use depended on continuous offerings being made to an individual. At the same time, however, direct requests for Adderall were largely avoided, as these were considered likely to give rise to uncomfortable situations—for example, a person making an explicit request might be perceived as greedy or presumptuous, or might risk putting a friend in the awkward position of having to refuse an appeal for help. Thus, more circumspect tactics were considered to be called for, particularly if one was hoping to receive pills on a regular basis.

During a discussion of how he acquires his medications, Jas, an undergraduate science major, offers a typical account of such a pattern of exchange, and alludes to ‘scrounging’ behaviours that are involved:

JAS: That first time, a friend gave me five 10-mg pills and I broke them in half, so it was ten days' worth and went from there.

INTERVIEWER: And that's how you get them now, the same way?

JAS: Well, I don't ask, like I did then. It's more scrounging around from the friends that I know have lots of extras. Like I said, people get prescribed significantly more than they actually use.

IV: So you don't normally ask them?

JAS: No. Asking all the time, that would be … I just make sure, I do what I can so that it’ll be offered.

Individuals reported a variety of similar ‘scrounging’ practices, directed towards individuals known to have supplies that might be shared, and performed in the hopes of yielding an offer of medication. Most commonly, informants reported dropping hints in conversations that could be picked up on by a receptive interlocutor. For example, Estelle explains the efforts she made after having received Adderall from a friend for the first time:

ESTELLE: What I did was, afterwards, I made it very clear that I was open to more. ‘Oh, you know, it was amazing, it made things so much easier—thank you so much! And you get to use it all the time, I'm so jealous!’ And she got the message. It was pretty obvious.

IV: But not as obvious as, say, just asking her?

ESTELLE: No. I didn't do that. I didn't know how she would feel, you know? And even now [after regular, ongoing exchanges], I just wouldn't. I just let her know, keep on telling her how great she is—and how great it is!

In addition to discussing benefits derived from Adderall, and voicing appreciation of gifts, individuals might also emphasize stresses or worries about difficult workloads. Anna explains how she does this deliberately, when attempting to elicit an offer from a friend:

ANNA: I'll just steer the conversation toward that, and say something like, ‘I don't know what to do, in the next 3 weeks I've got a paper for English, one for History, a report for Bio; and three killer finals, too. It's soooo much …’

In a similar vein, Jeff explains how to present an account of one's academic troubles in a manner likely to lead to an offer of assistance in the form of medication:

JEFF: You just make it clear that you’re in over your head, like, ‘I'm in deep shit here!’, and you drive it home. Eventually, if he's got some, he’ll feel bad keeping it to himself, when he knows he can help you out. If he can manage, he’ll offer you some. Or, at least, he'll say ‘sorry man, I've only got enough for me right now’, and then you know, you can move on and try elsewhere.

While the deliberate management of conversational exchanges was the most commonly reported scrounging strategy, some individuals described practices oriented toward fostering emotional and/or spatial closeness between donor and recipient. For example, Aiden tells me that he makes a point of being present with a friend at times when she is likely to be taking Adderall herself. Physical co-presence, a shared social experience, and feelings of togetherness among ‘study buddies’ leads to Adderall exchanging hands:

AIDEN: I don't ask, or even hint much, really. I just, I know the days she tends to be on it; and so I'm like, ‘Oh, hey, I'm going to hit the books today—what, you are too? Well, we should hang out!’ And when she's going to take some, she’ll just look at me and smile, and look at the bottle. And I'll shrug, sort of to say, ‘well, ok, sure I guess.’

Despite variations in these different tactics, this group of friendship-oriented, gift-seeking practices share in common significant features that are captured by Jas's felicitous use of the term ‘scrounging,’ above. They involve attempts to identify and subtly manipulate sources from which medication can be obtained for free; they are associated with a sense of uncertainty that is experienced in the face of a more or less unreliable supply; and they tend to reflect a dependence on the beneficence of others that must be actively encouraged. Moreover, informants often acknowledged that however subtle such strategies might be, they involved a manipulation of one's friends. While seldom relished, this was considered a necessary part of what needed to be done to obtain the pills that were sought.

### 2.2 Acquisition from family members

A related though less common set of practices (reported by nine participants) involved the acquisition of Adderall from family members or intimate partners. Exchanges of Adderall through family connections resembled gift transactions among friends, insofar as they took place in the absence of monetary exchange. However, family-oriented strategies often involved practices that would be regarded as unsuitable or unthinkable if directed toward friends. Most notably, informants indicated that the sort of explicit request for Adderall that was difficult to make to a friend could be made with relative ease to a family member.

In response to a question about how he obtains Adderall from a brother who uses it as a prescribed treatment for ADHD, for example, James explains why he feels able to make requests directly:

IV: So you just ask him for it, and he gives it to you?

JAMES: Yeah, of course. I mean, he has plenty, and it's not like he would be like, ‘no—it’s mine!’ He just wouldn't, we’re not like that. And it's his prescription, ok, but it's like, my Dad's insurance is what pays for it.

IV: And he doesn't have any problem … You know, even though you don't have ADHD?

JAMES: No. I mean, he knows that he takes it and it helps him with school. And he knows that if he gives it to me, it helps me with my stuff, too. What does it matter, who's got the diagnosis? It's like: you’ve got something, it helps, can help me. Of course you’re going to share.

For James, Adderall appears to be considered something akin to a collectively owned, family good. James suggests that even if the pills he receives are, in one sense, a gift from his brother, they are at the same time not really his brother's to give, since they are paid for by his family's medical plan. Moreover, as our conversation continued, it became apparent that James' receipt of Adderall was not accompanied by any significant sense of gratitude or reciprocal obligation. Sharing between family members was expected as a matter of course.

Other informants indicated that family members with legitimate prescriptions could be less willing to share than was James' brother; in fact, some even resisted demands placed upon them. However, in such cases the nature of the close ties between seeker and potential supplier seemed to allow direct strategies of manipulation to be used, without giving rise to the negative feelings associated with scrounging from friends. For example, Adam, a social science undergraduate, tells me that it's not a big deal for him to ‘guilt-trip’ his girlfriend of several years into giving up some of her prescribed supply. ‘She does get a little weirded out by it [a direct request], but she’ll eventually go along’, he says as he explains a typical situation in which he asks for some Adderall to help him through an end-of-semester crunch. ‘I’ll be, “Look, don't you want me to do well? I need it, I need to ace these. It's my future we’re talking about here.” And she’ll be, “Ok …”.’

Perhaps the clearest indication of the distinct quality of family-oriented acquisition strategies was the fact that practices extending beyond emotional manipulation could be entertained as options—including behaviours reported by two individuals that might be considered theft. Frieda, for instance, tells me that she obtains her study pills while returning to her family home during holidays or other breaks. During such visits, she surreptitiously goes into the family medicine cabinet and ‘skims’ pills from bottles of medication that are intended for her ADHD-diagnosed little sister. Although she does confess feeling guilty about such behaviour, Frieda nevertheless feels able to obtain Adderall from her sister in a way that she insists she would never consider in relation to a friend. When I ask her whether she has ‘skimmed’ pills from a friend's medicine cabinet, she appears shocked at the suggestion, emphatically telling me: “No way, I would never—that would be stealing. I mean, for real.” Similar to James, Frieda appears to regard a family member's supply of medication as something akin to communal property, a share of which one might more or less rightfully expect.

### 2.3 Acquisition from black market sources

Eight individuals reported paying cash for pills from those willing to sell them on what was, in essence, a black market existing within and around the university campus. Practices oriented to these sources diverged from those outlined above, insofar as they involved monetary exchange, and tended to involve more impersonal interactions as are generally associated with market-oriented transactions. This was especially apparent in cases that involved buying Adderall from individuals described as campus-based ‘drug dealers’: While the main trade of such dealers consisted of recreational street drugs such as marijuana and cocaine, they could also provide Adderall and other prescription drugs, through transactions that were almost entirely business-like in terms of their impersonality.

However, distinctions between market and non-market exchanges appeared much less clear-cut in relation to the most frequently reported black-market transactions; these (reported by seven individuals) involved the purchase of Adderall not from ‘dealers’, but from students who were situated within a buyer's extended social networks. Such suppliers tended to be described in familiar and relatively informal terms, for example as someone that one ‘sort of knows’, or as a ‘friend-of-a-friend.’ At the same time, however, the distance between exchanging partners was such that the employment of strategies used for acquiring Adderall as a gift would not be considered viable. Whereas friends might be approached with the hope of receiving pills for free, receipt of Adderall from acquaintances could only be expected in exchange for payment.

The acquisition of black market Adderall from acquaintances was not always easy, as connections to sellers were most often made through mutual friends. This meant that locating acquaintance-based sources would usually depend on the ability of a potential buyer to mobilise personal networks, as Jeff, indicates when explaining the strategy he uses when he is unable to obtain pills for free:

JEFF: I'll just ask around, ‘hey, I want to stock up a bit for end of term–do you know anyone who's got some that they'd be willing to part with?’ And they'll ask how much I'm willing to pay, and then ask around, if they know anyone who has it’

The nature of resulting transactions was somewhat ambiguous, insofar as these blurred distinctions between market and gift relations. Reliance upon friends who were willing to help find a source meant that buyers often experienced uncertainty about the reliability of their supplies; moreover, to the extent that successful acquisition were dependent upon the goodwill of connection-making friends, it might call for interpersonal efforts akin to those involved when scrounging gifts. At the same time, pills obtained from acquaintances could usually be purchased more cheaply than those bought from a dealer, with prices sometimes so low—for example, just enough to cover the costs that an acquaintance would have incurred at a pharmacy—as to be almost gift-like.

A notable feature of market-oriented acquisition strategies was their association with particular beliefs and understandings about what purchased Adderall might reveal about its users. While one might expect market-oriented exchanges to be a preferred means of acquisition, to the extent that they could offer more impersonal transactions and increased reliability of supplies, most individuals expressed a strong preference for gifted Adderall. Rather than being linked to concerns about financial cost, this preference was usually articulated in connection with the belief that someone who would go so far as buying pills might be too dependent on them; the implicit expectation being that one ought to be able to take Adderall or leave it, and only take it when it came for free. Purchased Adderall also tended to be associated with subtle but significant meanings that differentiated it from Adderall received as a gift: when purchased, it was something that seemed more like a ‘drug,’ with connotations of street use, danger, and outright illegality, and less like a ‘medication,’ which by comparison was perceived as relatively safe and socially accepted. Moreover, those who had never paid for the medication often emphasised that fact, in such a way as to imply a significant distinction between themselves and others who had.

### 2.4 Acquisition from deceived clinicians

A separate set of market-oriented acquisition strategies involved the extraction of Adderall for non-medical use from legal pharmaceutical markets, through means of clinical deception. Four individuals reported obtaining Adderall as a result of carefully-planned, fraudulent encounters with healthcare providers. These involved strategies oriented toward deliberately manipulating physicians in a way that would lead toward a (false) diagnosis of ADHD, which in turn would result in a prescription for the desired medication.

One such encounter was described by Lucy, a second-year undergraduate who explained to me how her plans had developed out of a visit to her doctor, regarding what she believed to be depression. Lucy had reported several psychiatric symptoms relating to low mood, but also one which led her clinician to raise the possibility of prescribing a stimulant medication:

LUCY: I told her about the problem, of feeling really down, and sometimes crying and stuff. But I also mentioned that I was having concentration problems—and she said, ‘oh it might be ADD [Attention-Deficit Disorder]. But, you know, we’re going to put you on anti-depressants first.’

Although Lucy did not consider there to be any chance that she actually had ADD, she had in fact already been using gifted Adderall to help her study. She thus seized upon her doctor's mention of ADD as an opportunity to access a new, more reliable source of Adderall. After a few months on antidepressants, she returned to her doctor with this plan in mind. ‘I just used that’, she explained, referring to the physician's prior speculation about ADD. ‘I said, oh yeah, well, you know, that anti-depressants are really working, but I'm still having trouble concentrating on my work.’ Believing it would ‘play better’ if she did not appear too eager, Lucy describes feigning resistance in response to the clinician's suggestion that her antidepressant be supplemented with an ADHD medication:

LUCY: I just played into the whole fact of being like [… ] nervous about taking the medication. And, you know, she kept soothing me and saying, “no, no, no, like, you know, lots of people take medication for it, it's absolutely fine, you know, we're just going to start you off low, we’ll see how it goes, and you know, see how you responded, you might not respond to it at first so if you need to increase it, you know, that's fine, we’ll talk about that—come back next week.”

Informing me that this was ‘exactly what I wanted’, Lucy went on to explain how, over a series of subsequent appointments, she successfully executed plans that resulted in a tripling of her initially prescribed dosage. This provided her with more pills than she herself regularly used, and thus with a surplus that she could sell to others on campus, in order to recoup the costs she incurred when paying to have her prescription filled at her pharmacy.

Three other individuals described similar approaches involving deliberate deception, although without any prior discussion of ADHD or stimulant medications with their doctors. Aaron, for example, told me about a strategy he developed after hearing ‘good things’ about non-medical Adderall use from those around him on campus. Feeling uncomfortable about requesting the drug from his friends, he conducted research online that led him to decide to try ‘faking’ ADHD symptoms. Preparations for deceiving his doctor included studying ADHD diagnosis criteria, and using a pen to scrawl a variety of notes and reminders on his hands immediately before his appointment, in the hope that these would be noticed as a symptom—he had learned that ‘people with ADD or whatever have trouble keeping track of stuff, they forget a lot.’ Following a successful encounter, Aaron left his doctor's office with an initial prescription for a month's supply, which was subsequently renewed on a regular basis.

While only four individuals engaged in this strategy, it is notable that they all reported using Adderall on a more frequent basis than did those who acquired the medication from other sources. No respondents reported taking Adderall on a daily basis for more than a few consecutive days, when receiving it as a gift or purchasing black market supplies. Yet all four of the individuals who obtained their own prescriptions did: two reported having previously taken the medication everyday for several weeks in a row before establishing their current patterns of intermittent use, while two reported patterns of daily use that had been maintained for more than three months. One reason for this tendency toward more regular use appeared to relate to the fact these individuals found themselves with a steady and ample supply of the medication. An illustration of the significance of reliability associated with prescription-sourced Adderall was provided by Marcus, who describes how his everyday decision-making process changed as he switched from acquiring Adderall irregularly from friends to obtaining it via his own prescription: ‘Before, it had been “should I take some today, or may be save it when I’ll need it more?” But now, it's more like, I ask myself: “why not take it?” Once a prescription holder, Marcus did not experience anxieties relating to medication scarcity that arose when scrounging for Adderall from friends, and felt able to take pills freely, without a need to consider rationing his supplies.

## 3 Discussion

The preceding analysis provides insights into the experiences, practices and perspectives of non-medical Adderall users, and results suggest that the means through which non-medical users obtain their supplies of medication are characterised by a significant degree of complexity and heterogeneity. This diversity is particularly striking if non-medical acquisition strategies are compared to the simplicity of how Adderall is obtained when legally prescribed to patients as a therapeutic intervention. Legitimately prescribed patients would generally proceed along a predictable pathway, taking a prescription obtained in the clinic to a pharmacy, where it would be exchanged for medication. In contrast, practices of drug acquisition among non-medical users of Adderall appear to be much less standardized, varying considerably in relation to the particular social and everyday circumstances in which individuals find themselves. Moreover, the use of different strategies for non-medical Adderall acquisition appears to correspond to subtle but significant differences in users' perceptions and experiences: Adderall bought from a ‘drug dealer,’ for example, is likely to be perceived differently from that received from a friend for free or from a doctor that has been tricked into providing it; and each different source of the medication is associated with a slightly different set of practical and ethical challenges with which an individual might be required to engage.

Findings of such diversity among different users is certainly consonant with well-established scholarship that indicates that the choices, behaviours and experiences of individual drug users vary according to the particular circumstances and local conditions within which individuals find themselves (Fraser and Moore, 2011). They also offer support for recent suggestions that prescription drug diversion is a complex and multi-faceted phenomenon, involving considerable variation between different classes of medication, and between different subpopulations of users (Fischer et al., 2010; Quintero et al., 2006). Moreover, such insights into the experiences, practices and perspectives of nonmedical stimulant users as provided above would seem necessary for developing an empirically-grounded understanding of the circulation and use of prescription stimulants among ‘healthy’ university students: It would be difficult to understand the relatively free movement and use of nonmedical Adderall among my informants, for example, without taking into account the practical strategies that make the substance accessible, and the particular understandings held by students that make the drug appear as something more or less acceptable to illicitly acquire and consume.

At the same time that the preceding results and analysis have explored the significance of local and everyday phenomena associated with drug diversion, it is important to note that the everyday beliefs and actions of individuals who seek, receive, and distribute unprescribed medications such as Adderall are also connected to larger cultural and political-economic dynamics—including those associated with the provision of national healthcare, scientific enterprise, and legal pharmaceutical markets. For example, as Lovell (2006) observes in relation to her study of buprenorphine diversion, the existence of medications as black market goods depends upon the medical knowledges and regulatory mechanisms that allow for the legal production and provision of those substances: these are required to make a pharmaceutical product's very existence possible, and in their absence there would be no legitimate commodity to divert from a legal market.

In relation to Adderall, the pharmaceutical markets that exist for the legal circulation of stimulant drugs can be considered to provide one of the most basic necessary conditions for students' illegal trades. Moreover, empirical evidence of a close interrelationship between legal markets and illicit use is suggested by recent research that has begun to identify correlations between the overall size of legal markets for prescription stimulants and rates of non-medical use of such drugs. Poulin (2007), for example, reports population-level findings from Canada that suggested that patterns of medical and non-medical use of prescription stimulants were closely connected with one another. Further support is also provided by findings of longitudinal survey research conducted by McCabe et al. (2014), which demonstrate that a decade-long increase in reports of medical use of prescription stimulants to treat ADHD correlated with a comparable increase in reported diversion behaviours and non-medical use.

It thus seems reasonable to suggest that the experiences and practises of the individuals reported above are intertwined with broader commercial and professional dynamics that studies of pharmaceuticalization have identified in relation to the growth of markets for ADHD drugs. These would include the economic and regulatory factors that have shaped ADHD as a clinical entity, and have produced a ‘diagnostic expansion’ in recent years that has allowed a wider range of individuals to qualify for legal access to prescription stimulants (Conrad and Potter, 2000). Within the US, they would also include the influence of Direct-to-Consumer advertisements, forms of consumer-group activism, and other initiatives financed by pharmaceutical companies that may directly or indirectly promote medication use, and also reinforce perceptions of psycho-stimulant pharmaceuticals as relatively safe, health-enhancing substances (Cohen, 2006; Loe and Cuttino, 2008).

### 3.1 Limitations of current study

The findings presented above reflect limitations that often arise in relation to qualitative and naturalistic research, particularly when conducted on a relatively small scale. While providing rich information pertaining to understandings of and interactions between individuals, the fact that research was conducted on only one university campus make it difficult to stake convincing claims about generalizability of results in relation to other contexts, both within and beyond the US. Moreover, use of semi-structured interviews raises the potential for unintended interview effects, whereby informants may offer what they consider to be socially desirable responses, rather than truthful ones. Finally, the use of non-randomised sampling procedures means that the population under study cannot be taken as representative of all students at the university where the study took place, and also raises the possibility of self-selection bias.

## 4 Conclusion

This article has presented findings that add to existing knowledge about prescription drug diversion, by elucidating some of the everyday practises and understandings that arise in relation to the non-medical use of stimulant medications among university students—a population that has been associated with particularly pronounced levels of such use (Quintero et al., 2006). It has shown that significant divergences in users' practices and experiences of nonmedical stimulant use arise in relation to acquisition strategies employed by individuals, as these relate to the four sources of illicit medication supplies that were most commonly reported (i.e., friends; family members; black market dealers; deceived clinicians). It has further shown that different acquisition strategies correspond to variations in how individuals think and feel about their nonmedical drug usage. Combined, the reported findings suggest that how and why prescription stimulants are used may vary significantly between individuals, even when they might superficially appear to constitute a single, homogenous population (e.g., university students consuming stimulants for the non-medical purpose of improving academic performance). Such findings are significant because they indicate that nonmedical prescription stimulant is a phenomenon that involves a greater degree of complexity than might be apparent from consideration of statistical or population-level data.

While findings relating to the particularities of users' everyday experiences and practises might be dismissed as relatively unimportant details by those experts and agencies that seek to address public health issues associated with nonmedical prescription drug use, such a dismissal would be misguided. Indeed, if social circumstances and individual contingencies do in fact constitute significant factors in determining how and why students come to take prescription stimulants, then a better understanding of these would be helpful in relation to the consideration of ethical, regulatory, and public health questions arising in relation to nonmedical stimulant use. As Quintero et al. (2006) note, surveillance data and media reports suggest significant trends in prescription drug misuse among university students, yet little is known about social and cultural factors that shape individuals' practices. This dearth of knowledge is significant, because detailed understandings of how drug diversion happens within different groups of users might be of significant value in relation to education and prevention initiatives that seek to change individual behaviours, particularly if these are best developed in such a way that takes individual-level variations into account (Wilens et al., 2008). For example, findings above might be useful in devising initiatives that are specifically oriented to different practices of drug diversion that arise in relation to different sources of supply and related acquisition strategies.

It is worth reiterating that the study reported here was conducted at a single university, and therefore provides findings that are limited in their generalizability. Nevertheless, results suggest fruitful avenues for further research on non-medical prescription stimulant use. Future studies might pursue more systematically the findings reported above that suggest that divergences in the acquisition strategies that are employed by individuals correspond to differences in how users think and feel about Adderall. More broadly, findings above suggest that further use of sociocultural research strategies could increase understandings of how drug diversion happens among the individuals and groups who actively engage in the illicit use and exchange of pharmaceutical products.

A further avenue for research would involve exploring in more detail the potential of Lovell's concept of ‘pharmaceutical leakage’ for contributing to studies of ‘pharmaceuticalization’ that examine the expanding usage of medications within a wide range of personal and socio-medical contexts (Bell and Figert, 2012)—and particularly those which seek to understand pharmceuticalization as a complex, multi-faceted social and medical process (Williams et al., 2011). Lovell develops her ideas exclusively in relation to buprenorphine users in France, with little discussion of other medications. However, the analysis above suggests that her framework has applicability to cases beyond that of buprenorphine; it may be particularly useful in extending analyses of how individual and consumer experiences intersect with broader political and economic dynamics (Fox et al., 2005; Jones, 2008; Stevenson et al., 2008). It has been suggested that recent studies of pharmaceutical research, development and commerce (e.g., Petryna, 2009; Pollock, 2012; Wahlberg and McGoey, 2007) indicate that the pharmaceutical industry is a complex and multi-faceted entity akin to a massive elephant whose form can only be grasped in parts at a time (Dumit, 2012: 18). With its orientation toward investigating how globally produced and marketed products enter into the circuits of everyday use, and how effects and meanings of pharmaceuticals are mediated by the personal and social circumstances of individual who consume them, Lovell's approach might allow for the dynamics of pharmaceutical industry to be understood in even greater complexity, as extending down into the everyday worlds of those who consume the industry's products.

## References

[bib1] Arria A.M. (2008). Nonmedical use of prescription stimulants and analgesics: associations with social and academic behaviors among college students. J. Drug Issues.

[bib2] Bazeley P. (2007). Qualitative Data Analysis with NVivo.

[bib3] Bell S., Partridge B., Lucke J., Hall W. (2013). Australian university students' attitudes towards the acceptability and regulation of pharmaceuticals to improve academic performance. Neuroethics.

[bib4] Bell S.E., Figert A.E. (2012). Medicalization and pharmaceuticalization at the intersections: looking backward, sideways and forward. Soc. Sci. Med..

[bib5] Bond J. (2009). Stages in cognitive impairment: boundary issues and the biomedicalisation of later life. Gerontologist.

[bib6] Charmaz K. (2006). Constructing Grounded Theory: A Practical Guide through Qualitative Analysis.

[bib7] Cohen D., Lloyd G., Stead J., Cohen D. (2006). Critiques of the ‘ADHD’enterprise. Critical New Perspectives on ADHD.

[bib8] Conrad P., Leiter V. (2004). Medicalization, markets and consumers. J. Health Soc. Behav..

[bib9] Conrad P., Potter D. (2000). From hyperactive children to ADHD adults: observations on the expansion of medical categories. Soc. Probl..

[bib10] Coveney C., Gabe J., Williams S. (2011). The sociology of cognitive enhancement: medicalisation and beyond. Health Sociol. Rev..

[bib11] Dumit J. (2012). Drugs for Life: How Pharmaceutical Companies Define Our Health.

[bib12] Elnicki D.M. (2013). Cognitive enhancement drug use among medical students and concerns about medical student well-being. J. General Intern. Med..

[bib13] Farah M.J., Illes J., Cook-Deegan R., Gardner H., Kandel E., King P. (2004). Neurocognitive enhancement: what can we do and what should we do?. Nat. Rev. Neurosci..

[bib14] Fischer B., Bibby M., Bouchard M. (2010). The global diversion of pharmaceutical drugsnon-medical use and diversion of psychotropic prescription drugs in North America: a review of sourcing routes and control measures. Addiction.

[bib15] Fox N.J., Ward K.J., O'Rourke A.J. (2005). The ‘expert patient’: empowerment or medical dominance? The case of weight loss, pharmaceutical drugs and the Internet. Soc. Sci. Med..

[bib16] Fraser S., Moore D. (2011). The Drug Effect: Health, Crime and Society.

[bib17] Greely H., Sahakian B., Harris J., Kessler R.C., Gazzaniga M., Campbell P. (2008). Towards responsible use of cognitive-enhancing drugs by the healthy. Nature.

[bib18] Hall K.M., Irwin M.M., Bowman K.A., Frankenberger W., Jewett D.C. (2005). Illicit use of prescribed stimulant medication among college students. J. Am. Coll. Health.

[bib19] Hotze T.D., Shah K., Anderson E.E., Wynia M.K. (2011). “Doctor, would you prescribe a pill to help me…?” A national survey of physicians on using medicine for human enhancement. Am. J. Bioeth..

[bib20] Jones K. (2008). In whose interest? Relationships between health consumer groups and the pharmaceutical industry in the UK. Sociol. Health Illn..

[bib21] Kramer P.D. (1992). Listening to Prozac.

[bib22] Loe M. (2008). The prescription of a new generation. Contexts.

[bib23] Loe M., Cuttino L. (2008). Grappling with the medicated self: the case of ADHD college students. Symb. Interact..

[bib24] Lovell A.M., Petryna A., Lakoff A., Kleinman A. (2006). Addiction markets: the case of high-dose buprenorphine in France. Global Pharmaceuticals: Ethics, Markets, Practices.

[bib25] Lucke J.C. (2012). Empirical research on attitudes toward cognitive enhancement is essential to inform policy and practice guidelines. AJOB Prim. Res..

[bib26] Maher B. (2008). Poll results: look who's doping. Nature.

[bib27] McCabe S.E., Knight J.R., Teter C.J., Wechsler H. (2005). Non-medical use of prescription stimulants among US college students: prevalence and correlates from a national survey. Addiction.

[bib28] McCabe S.E., West B.T., Teter C.J., Boyd C.J. (2014). Trends in medical use, diversion, and nonmedical use of prescription medications among college students from 2003 to 2013: connecting the dots. Addict. Behav..

[bib29] Moreira T., May C., Bond J. (2009). Regulatory objectivity in action: mild cognitive impairment and the collective production of uncertainty. Soc. Stud. Sci..

[bib30] Parens E. (1998). Enhancing Human Traits : Ethical and Social Implications.

[bib31] Partridge B., Bell S., Lucke J., Hall W. (2013). Australian university students' attitudes towards the use of prescription stimulants as cognitive enhancers: perceived patterns of use, efficacy and safety. Drug alcohol Rev..

[bib32] Petryna A. (2009). When Experiments Travel: Clinical Trials and the Global Search for Human Subjects.

[bib33] Pilkinton M., Cannatella A. (2012). Nonmedical use of prescription stimulants: age, race, gender, and educational attainment patterns. J. Hum. Behav. Soc. Environ..

[bib34] Pollock A. (2012). Medicating Race: Heart Disease and Durable Preoccupations with Difference.

[bib35] Poulin C. (2007). From attention-deficit/hyperactivity disorder to medical stimulant use to the diversion of prescribed stimulants to non-medical stimulant use: connecting the dots. Addiction.

[bib36] Quintero G., Nichter M., Singer M., Erickson P.I. (2011). Generation RX: anthropological research on pharmaceutical enhancement, lifestyle regulation, self-medication and recreational drug use. A Companion to Medical Anthropology.

[bib37] Quintero G., Peterson J., Young B. (2006). An exploratory study of socio-cultural factors contributing to prescription drug misuse among college students. J. Drug Issues.

[bib38] Rudski J.M. (2014). A comparison of attitudes toward pharmacological treatment versus enhancement under competitive and noncompetitive conditions. AJOB Empir. Bioeth..

[bib39] Singh I., Kelleher K.J. (2010). Neuroenhancement in young people: proposal for research, policy, and clinical management. AJOB Neurosci..

[bib40] Singh I., Kendall T., Taylor C., Mears A., Hollis C., Batty M. (2010). Young people's experience of ADHD and stimulant medication: a qualitative study for the NICE guideline. Child Adolesc. Ment. health.

[bib41] Stevenson F.A., Leontowitsch M., Duggan C. (2008). Over-the-counter medicines: professional expertise and consumer discourses. Sociol. Health Illn..

[bib42] Teter C.J., McCabe S.E., LaGrange K., Cranford J.A., Boyd C.J. (2006). Illicit use of specific prescription stimulants among college students: prevalence, motives, and routes of administration. Pharmacotherapy.

[bib43] Varga M.D. (2012). Adderall abuse on college campuses: a comprehensive literature review. J. Evidence-Based Soc. Work.

[bib44] Vrecko S. (2013). Just how cognitive is “cognitive enhancement”? On the significance of emotions in university students' experiences with study drugs. AJOB Neurosci..

[bib45] Wahlberg A., McGoey L. (2007). An elusive evidence base: the construction and governance of randomized controlled trials. Biosocieties.

[bib46] Whitehouse P., Gaines A.D., Lindstrom H., Graham J.E. (2005). Anthropological contributions to the understanding of age-related cognitive impairment. Lancet Neurol..

[bib47] Wilens T.E., Adler L.A., Adams J., Sgambati S., Rotrosen J., Sawtelle R. (2008). Misuse and diversion of stimulants prescribed for ADHD: a systematic review of the literature. J. Am. Acad. Child Adolesc. Psychiatry.

[bib48] Williams S.J., Martin P., Gabe J. (2011). The pharmaceuticalisation of society? A framework for analysis. Sociol. health & Illn..

